# Evaluation of force released by deflection of orthodontic wires in conventional and self-ligating brackets

**DOI:** 10.1590/2177-6709.21.6.091-097.oar

**Published:** 2016

**Authors:** Rodrigo Hitoshi Higa, Nayara Thiago Semenara, José Fernando Castanha Henriques, Guilherme Janson, Renata Sathler, Thais Maria Freire Fernandes

**Affiliations:** 1 PhD resident in Orthodontics, School of Dentistry, Universidade de São Paulo, Bauru/SP, Brazil.; 2 DDS, School of Dentistry, University of São Paulo, Bauru/SP, Brazil.; 3 Full professor, Department of Orthodontics, School of Dentistry, Universidade de São Paulo, Bauru/SP , Brazil.; 4 Professor, Hospital for Rehabilitation of Craniofacial Anomalies, Universidade de São Paulo, Bauru/SP , Brazil.; 5 Full professor, Universidade Norte do Paraná, Londrina/PR, Brazil.

**Keywords:** Orthodontic wires, Orthodontic brackets, Comparative study, Mechanical phenomena

## Abstract

**Introduction:**

The aim of the study was to evaluate deflection forces of rectangular orthodontic wires in conventional (Morelli^TM^), active (In-Ovation R^TM^) and passive (Damon 3MX^TM^) self-ligating brackets.

**Material and Methods:**

Two brands of stainless steel and nickel-titanium (NiTi) wires (Morelli^TM^ and GAC^TM^), in addition to Ormco^TM^ copper-nickel-titanium wires were used. Specimens were assembled in a clinical simulation device especially designed for this study and tested in an Instron universal testing machine. For the testing procedures, an acrylic structure representative of the maxillary right central incisor was lingually moved in activations of 0 to 1 mm, with readings of the force released by deflection in unloading of 0.5, 0.8 and 1 mm at a constant speed of 2 mm/min. Inter-bracket forces with stainless steel, NiTi and CuNiTi were individually compared by two-way ANOVA, followed by Tukey’s tests.

**Results:**

Results showed that there were lower forces in conventional brackets, followed by active and passive self-ligating brackets. Within the brands, only for NiTi wires, the Morelli^TM^ brand presented higher forces than GAC^TM^ wires.

**Conclusions:**

Bracket systems provide different degrees of deflection force, with self-ligating brackets showing the highest forces.

## RESUMO

**Objetivo:**

o objetivo deste estudo foi avaliar as forças de deflexão de fios ortodônticos retangulares em braquetes convencionais (Morelli^TM^) e autoligáveis ativos (In-Ovation R^TM^ ) e passivos (Damon 3MXT^M^).

**Material e Métodos:**

foram utilizadas duas marcas comerciais (Morelli^TM^ e GAC^TM^) de fios de aço inoxidável e de níquel-titânio (NiTi), além do fio de NiTi com adição de cobre (Ormco^TM^). Os espécimes foram montados em um dispositivo de simulação clínica especialmente desenhado para esse estudo e testado em uma máquina universal de ensaios Instron. Para o procedimento dos testes, a peça representativa do incisivo central superior direito foi movida no sentido vestibulolingual em ativações de 0 a 1 mm, com leituras da força liberada pela deflexão em 0,5; 0,8 e 1 mm, em uma velocidade constante de 2 mm/min. As forças interbraquetes com os fios de aço, de NiTi e NiTi com adição de cobre foram individualmente comparadas pelo teste de ANOVA a dois critérios, seguido pelo teste de Tukey.

**Resultados:**

houve menor liberação de forças nos braquetes convencionais, seguidos pelos braquetes autoligáveis ativos e passivos. Entre as marcas comerciais, somente houve diferença para o fio de NiTi, onde a marca Morelli^TM^ apresentou maiores forças do que a GAC^TM^.

**Conclusão:**

os braquetes promovem diferentes graus de forças de deflexão, sendo que os braquetes autoligáveis liberam as maiores forças.

## INTRODUCTION

In Orthodontics, light and continuous force exerted to obtain controlled tooth movement has been accepted as ideal.^1^ Orthodontic wires for leveling and alignment must be able to release such forces and transmit them in a broad range of activation. One of the alloys with such a feature is the nickel-titanium (NiTi) one, which has advantageous characteristics; for instance, good elasticity and low stiffness, compared to stainless steel wire. These factors make it interesting for the early stages of treatment. With the development of metallurgy, NiTi wires with properties of improved superelasticity and shape memory have been created.

In the mid-90s, copper-added NiTi wires (CuNiTi) appeared in the market. They consist of nickel, titanium, copper and chromium. According to the manufacturer, due to incorporation of copper, those wires have more thermoactive properties than superelastic NiTi wires and allow acquisition of an optimal force system with a more precise control of tooth movement, thus enabling quantification and application of load levels appropriate to orthodontic treatment purposes.^2^ ^,^ ^3^ Choosing the best treatment protocol for patients, with efficient results and without causing damage to patients, ensures treatment success. Additionally, the correct wire sequence is a very important factor in this regard.

Although the right wire choice is very important, it is known that friction between wire and bracket and between wire and the ligation system may adversely affect the force released to patient’s teeth, thereby decreasing it.^4^ ^,^ ^5^ ^,^ ^6^ In orthodontic routine, the use of self-ligating brackets has become common. Manufactures claim several advantages in using these accessories; among them, low friction seems to be the most studied. Some studies confirm that in these brackets there is lower friction, thus increasing arch leveling efficiency and resulting in shorter treatment time.^7^ ^,^ ^8^ However, more studies and clinical evaluations are still necessary to ensure such benefits. According to the pressure the system applies to the orthodontic wire, self-ligating brackets may be: active, when the system presses the wire into the slot; or passive, when the system allows freedom of the wire inside the slot. Depending on the pressure that the self-ligating system applies to the wire, it is possible to obtain higher or lower friction, which can change the amount of force released for orthodontic movement. When using an active bracket system, friction is greater than when using a passive one.^9^ ^,^ ^10^ ^,^ ^11^

Given the variety of wire types, brackets and manufacturers, it is necessary to know their features for better application in the orthodontic clinic. Thus, this work aimed to study one of the factors that influences treatment efficiency: the force released by deflection of orthodontic wires routinely used in the orthodontic clinic, associated to conventional and self-ligating brackets.

## MATERIAL AND METHODS

### Experimental groups

Three sets of brackets were selected for this study: conventional Morelli (Dental Morelli^TM^, São Paulo, Brazil), active self-ligating (In-Ovation R, GAC^TM^, Bohemia, NY, USA) and passive self-ligating (Damon 3MX, Ormco^TM^, Orange, Calif., USA). Three different wire alloys were tested: stainless steel and NiTi (Morelli^TM^ and GAC^TM^), and CuNiTi (Ormco^TM^). In total, 15 groups were formed and, in each group, ten specimens were tested. The brackets used were preadjusted with a slot width of 0.022-in (0.56 mm). The wires were deflected from 0 to 1 mm. All wires tested were of 0.019 x 0.025-in rectangular section (Table 1). 

**Table 1:** Materials used in the study .

Bracket system	Alloy type	Wire diameter	Wire brand
Conventional Morelli	Stainless Steel	0.019 x 0.025-inch	Morelli
Conventional Morelli	Stainless Steel	0.019 x 0.025-inch	GAC
Conventional Morelli	NiTi	0.019 x 0.025-inch	Morelli
Conventional Morelli	NiTi	0.019 x 0.025-inch	GAC
Conventional Morelli	CuNiTi	0.019 x 0.025-inch	Ormco
In Ovation R	Stainless Steel	0.019 x 0.025-inch	Morelli
In Ovation R	Stainless Steel	0.019 x 0.025-inch	GAC
In Ovation R	NiTi	0.019 x 0.025-inch	Morelli
In Ovation R	NiTi	0.019 x 0.025-inch	GAC
In Ovation R	CuNiTi	0.019 x 0.025-inch	Ormco
Damon 3MX	Stainless Steel	0.019 x 0.025-inch	Morelli
Damon 3MX	Stainless Steel	0.019 x 0.025-inch	GAC
Damon 3MX	NiTi	0.019 x 0.025-inch	Morelli
Damon 3MX	NiTi	0.019 x 0.025-inch	GAC
Damon 3MX	CuNiTi	0.019 x 0.025-inch	Ormco

The wires were tied to conventional brackets through ring-shape elastomeric ligatures and to self-ligating brackets, according to their system: passive (Damon 3MX^TM^) or active (In-Ovation R^TM^). Wires, brackets and elastomeric ligatures belonged to the same batch.

### Deflection test

The tests of wire deflection force release were performed in a clinical simulation device representing all 14 maxillary teeth.^12^ ^-^ ^14^

**Figure 1:**
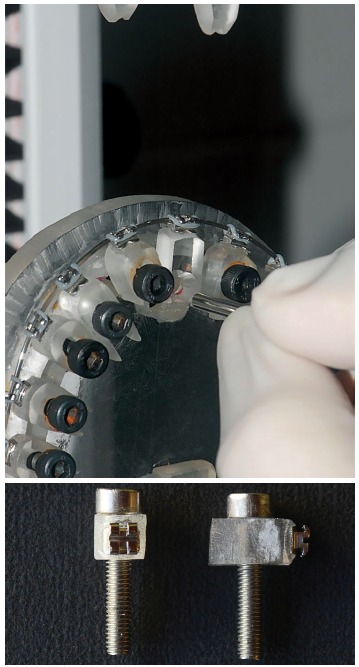
A) The clinical simulation device used in this study. B) block representative of the teeth connected to the screw.

The clinical simulation device consisted of an acrylic resin plate to which acrylic blocks representing maxillary teeth were fixed (Fig 1A). Brackets were bonded with cyanoacrylate ester gel (Super Bonder, Loctite) onto the acrylic blocks (Fig 1B). These blocks were fixed by means of screws inserted in the bottom of the acrylic resin plate. They were fixed to the acrylic plate, respecting a standard distance of 6 mm between brackets, since the force-deflection relation is dependent, among other things, on this distance. The parabola shape was determined by the wire to be tested, reducing the risk of diverse forces arising from deflection applied in an unexpected way.

**Figure 2:**
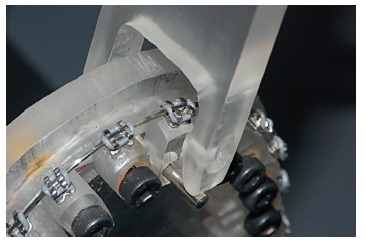
Tip of the universal testing machine moving bucco-lingually the acrylic structure.

Tests were performed on the block corresponding to the maxillary right central incisor. Unlike the others, this block was not screwed, enabling its bucco-lingual movement. It had a lingual perforation, in which a metal cylinder was placed, thus allowing its activation. The edge of the activation device, attached to the testing machine, had a rounded cut to fit the metal cylinder. The blocks were fixed to the clinical simulation device with the originally aligned arch, so the action line of the activating force acted perpendicularly to the plane of the bracket (Fig 2). The speed of the testing machine was 2 mm/min, in accordance with ISO 15841. 

Records of the force released by wire deflection were made in unloadings of 0.5 mm, 0.8 mm and 1 mm. The deflection tests were performed with the Instron universal testing machine with a load cell of 10 N.

### Statistical analyses

Descriptive statistics, including means and standard deviations, were calculated for each archwire-bracket combination. Normal distribution of variables was assessed by means of Kolmogorov-Smirnov tests. All variables showed normal distribution. Therefore, inter-bracket forces with stainless steel, NiTi and CuNiTi were individually compared by means of two-way ANOVA, followed by Tukey’s tests.

All statistical analyses were performed with Statistica software (Version 6.0, Statsoft, Tulsa, Okla., USA). Results were considered statistically significant at *p*< 0.05.

## RESULTS

Results were recorded in cN at deflections of 0.5, 0.8 and 1 mm. The results are shown in Tables 2 to 4 and divided according to the alloy used.

**Table 2:** Inter-bracket force (cN) comparison with stainless steel wire, in progressive deflections (Two-way Anova, followed by Tukey tests).

Elastic deflection	Morelli		In Ovation R		Damon 3MX		Bracket	Wire brand
	Morelli	GAC	Morelli	GAC	Morelli	GAC		
	Mean (SD)	Mean (SD)	Mean (SD)	Mean (SD)	Mean (SD)	Mean (SD)	P	P
0.5 mm	264.77	252.03	543.28	505.04	870.83	906.13	0.000*	0.867
	(92.18)^A^	(46.09)^A^	(31.38)^B^	(27.45)^B^	(62.76)^C^	(19.61)^C^		
0.8 mm	348.13	339.31	892.40	844.35	-----	-----	0.000*	0.161
	(99.04)^A^	(51.97)^A^	(20.59)^B^	(54.91) ^B^				
1.0 mm	396.18	396.18	-----	-----	-----	-----		0.070
	(105.91)	(55.89)

* Statistically significant at *p* < 0.05.

------: Values above 1000cN.

**Table 3:** Inter-bracket force (cN) comparison with NiTi wire, in progressive deflections (Two-way Anova, followed by Tukey tests).

Elastic deflection	Morelli		In ovation R		Damon 3MX		Bracket	Wire brand
	Morelli	GAC	Morelli	GAC	Morelli	GAC		
	Mean (SD)	Mean (SD)	Mean (SD)	Mean (SD)	Mean (SD)	Mean (SD)	P	P
0.5 mm	238.30 (43.48)^A^	160.82 (23.57)^B^	293.21 (55.52)^C^	237.32 (41.84)^A^	462.87 (24.74)^D^	401.09 (14.30)^E^	0.000*	0.000*
0.8 mm	304,2 (47.07)^A^	210.84 (25.33)^B^	457.97 (77.03)^C^	237.32 (45.27)^D^	714.9 (31.71)^E^	656.06 (19.60)^E^	0.000*	0.000*
1.0 mm	341.27 (49.72)^A^	246.14 (28.62)^B^	564.86 (85.11)^C^	465.81 (45.10)^D^	862.98 (32.67)^E^	813.95 (25.48)^E^	0.000*	0.000*

* Statistically significant at *p* < 0.05.

**Table 4:** Inter-bracket force (cN) comparison with CuNiTi wire, in progressive deflections (Two-way Anova, followed by Tukey tests).

Elastic deflection	Morelli	In ovation R	Damon 3MX	p
	Mean (SD)	Mean (SD)	Mean (SD)	
0.5 mm	253.01 (53.93)^A^	302.04 (42.16)^B^	419.72 (30.40)^C^	0.000*
0.8 mm	315.77 (62.76)^A^	438.35 (81.20)^B^	557.99 (32.36)^C^	0.000*
1.0 mm	349.11 (63.74)^A^	507.98 (89.64)^B^	582.51(38.24)^C^	0.000*

* Statistically significant at *p* < 0.05.

### A) Stainless steel

Stainless steel wires in conventional Morelli^TM^ brackets released significantly lower force, followed by active and passive brackets (Table 2). There were no statistically significant differences for Morelli^TM^ and GAC^TM^ stainless steel wires for the different brackets in different deflections. With a deflection of 0.8 mm for the passive and 1.0 mm for active and passive brackets, it was not possible to measure the forces released because they were greater than the load cell used (10 N).

### B) NiTi

The NiTi wires tested in conventional brackets released significantly lower forces, followed by active and passive brackets (Table 3). Regardless of the brackets used, in activations of 0.5, 0.8 and 1 mm, Morelli^TM^ wires showed significantly higher forces than GAC^TM^ wires, except when compared with Damon^TM^ ones in 0.8 and 1mm of deflection, in which they showed statistical similarity (Table 3).

### C) CuNiTi

Similarly, the CuNiti wires tested in conventional brackets released significantly lower forces, followed by active and passive brackets (Table 4).

## DISCUSSION

There are basically two ways of performing deflection tests: the 3-point test and the use of a clinical simulation device. As the objective of this study was to clinically simulate the behavior of wires and brackets, we chose to use a clinical simulation device representing all teeth. The clinical simulation device was based on previous works that had employed it.^12^ ^-^ ^14^

In this study, the wires were tested in dry conditions. The use of artificial saliva during testing is still controversial. Some authors believe that it may be important,^15^ ^,^ ^16^ while others think that the use of artificial saliva is not valid for laboratory tests because it does not adequately replace the human saliva.^17^ ^,^ ^18^ A previous study showed that the presence of human saliva has an inconsistent friction effect in sliding tests:^18^ sometimes the saliva acts as a lubricant, whereas in others it increases friction. Because of this controversy, we chose to test the wires in a dry environment.

The results will be discussed according to the alloys tested in this sequence: stainless steel, NiTi and CuNiTi in the three bracket systems. 

### Stainless steel wires

It was possible to notice the high loads released by stainless steel wires, especially in the 0.8 mm deflection for the passive and 1 mm of deflection for both self-ligating systems, ​​reaching values ​​higher than 10 N (Table 2). The forces with stainless steel wires increased from conventional to active and passive brackets, in order. The greatest force released by wires in self-ligating brackets probably result from the properties of stainless steel wires, the diameter used, and mainly the ligation method, which allows more freedom for the wires inside the slot, especially in the case of passive self-ligating brackets, thus decreasing friction between wire and ligation system.^7^ ^,^ ^19^

There was no force difference among wire brands. It seems that the manufacturing procedure of steel wires, as well as the quality of the material used is similar in the two companies.

### NiTi wires

In this study, the wires showed an increasing force trend when self-ligating brackets were used, similar to a previous study^12^ (Table 3). Contrastingly, another study using NiTi wires with a 0.016-in diameter, deflection of 2 mm and the 3-point test, found that elastomeric ligatures may limit the superelasticity of NiTi wires.^4^ Unlike the trend observed in this study, the authors found lower mean values ​​for self-ligating brackets (585 cN) and higher values ​​for brackets tied with metallic (783 cN) and elastomeric ligatures (638 cN). This difference may be related to the methodology, the diameter of the wire tested, elastomeric ligature brands, the self-ligating brackets used and the amount of deflection performed by the authors. Although friction influences the forces released by the wires, there are other factors that also play a role, such as arch dimension, amount of deflection, ligation method and frictional forces.^12^ ^,^ ^17^ ^,^ ^20^ The elastomeric ligatures used may have interfered with the seating forces, resulting in higher released forces for this ligation type. Other studies also found this effect of elastomeric ligatures.^21^ ^,^ ^22^ ^,^ ^23^ In this study, there was little influence of this factor, probably due to the amount of deflection and the diameter of the wires tested.

For conventional and active brackets, with deflections of 0.5, 0.8 and 1 mm, Morelli^TM^ wires had significantly higher forces than GAC^TM^ (Table 3). With passive brackets, Morelli^TM^ wires also showed higher force; however; only with 0.5 mm of deflection they were statistically significant (Table 3). Therefore, for NiTi wires, there is a difference in the force released between brands, and the orthodontist must be aware of this detail. A study compared 48 superelastic NiTi wires of five different brands and found wide variation in the behavior of these products, since some wires showed no superelastic characteristics.^24^ Some wires showed permanent deformation in the 3-point test. Another study also found significant differences in the forces released when comparing different brands of NiTi wires.^25^

### CuNiTi wires

Conventional brackets released the lowest forces while passive brackets released the greatest force in all tests (Table 4). By evaluating the forces released on premolars, another study^3^ using CuNiTi wires with 0.014 x 0.025-in dimensions, found no difference between the evaluated brackets (Orthos 2^TM^; Damon 2^TM^ and In Ovation^TM^). However, the forces released in 1mm of deflection for In Ovation^TM^ and Damon 2^TM^ brackets were very similar to those found in this study with wires of greater diameter. However, care must be taken in this comparison due to the use of different methods.

## CLINICAL CONSIDERATIONS

The current results lead to an interesting debate on the choice of brackets to be used in orthodontic treatment. Self-ligating brackets released greater forces than conventional ones. Friction appears to be responsible for this result, since it decreases the force during unloading.^24^ ^,^ ^26^ ^,^ ^27^ Passive brackets allow greater freedom of the wire inside the slot, reducing friction and releasing higher forces, as demonstrated. However, these forces can be considered high if brackets are combined with wires which provide high forces, such as larger-diameter or stainless steel wires. Accentuated forces cause hyalinization and necrosis of the neighboring tissue, greater pain sensation to the patient and increased risk of root resorption.^28^ During this time, tooth movement decreases or even stops, delaying treatment.^1^

The ideal is to combine the bracket system that promotes greater forces with wires that release lower forces. Superelastic wires promote this type of force, with the advantage of releasing these forces continuously, optimizing tooth movement. Copper-added wires appear to exert this function very well, as demonstrated in this study. However, the combination of these wires with brackets which deliver lower forces, as conventional brackets, can release suboptimal forces because these wires may be unable to overcome the friction generated by the ligature, thus providing a very slow orthodontic movement^29^ or even not producing movement.^30^ Thus, one can choose the bracket system that produces less friction, with greater dissipation of forces associated with superelastic wires that release lower forces, or brackets that have greater friction with wires which compensate this factor, producing higher forces. The advantage of the first choice is that the force released by the superelastic wire is continuous, and many reports indicate that continuous forces of low magnitude are more effective for tooth movement.^26^ ^,^ ^31^ ^-^ ^38^

## CONCLUSIONS

» Conventional brackets showed the lowest deflection forces, followed by active and passive self-ligating brackets, which showed the largest forces.

» There were differences between deflection forces released by different wire brands only for nickel-titanium archwires, with no difference for stainless steel ones. 
